# Human Echinococcosis Mortality in the United States, 1990–2007

**DOI:** 10.1371/journal.pntd.0001524

**Published:** 2012-02-07

**Authors:** Benjamin N. Bristow, Sun Lee, Shira Shafir, Frank Sorvillo

**Affiliations:** 1 Los Angeles County Department of Public Health, Los Angeles, California, United States of America; 2 Preventive Medicine Residency Program, California Department of Public Health, Sacramento, California, United States of America; 3 Department of Epidemiology, School of Public Health, University of California Los Angeles, Los Angeles, California, United States of America; 4 College of Health Sciences, Trident University International, Cypress, California, United States of America; Universidad Nacional Autónoma de México, Mexico

## Abstract

**Background:**

Despite the endemic nature of *Echinococcus granulosus* and *Echinococcus multilocularis* infection in regions of the United States (US), there is a lack of data on echinococcosis-related mortality. To measure echinococcosis-associated mortality in the US and assess possible racial/ethnic disparities, we reviewed national-death certificate data for an 18-year period.

**Methodology/Principal Findings:**

Echinococcosis-associated deaths from 1990 through 2007 were identified from multiple-cause-coded death records and were combined with US census data to calculate mortality rates. A total of 41 echinococcosis-associated deaths occurred over the 18-year study period. Mortality rates were highest in males, Native Americans, Asians/Pacific Islanders, Hispanics and persons 75 years of age and older. Almost a quarter of fatal echinococcosis-related cases occurred in residents of California. Foreign-born persons accounted for the majority of echinococcosis-related deaths; however, both of the fatalities in Native Americans and almost half of the deaths in whites were among US-born individuals.

**Conclusions/Significance:**

Although uncommon, echinococcosis-related deaths occur in the US. Clinicians should be aware of the diagnosis, particularly in foreign-born patients from *Echinococcus* endemic areas, and should consider tropical infectious disease consultation early.

## Author Summary

Human echinococcosis is a parasitic disease that affects an estimated 2–3 million people and results in an annual monetary loss of over $750,000,000 worldwide. It results in the development of life threatening tissue cysts, primarily in the liver and lung, following accidental ingestion of eggs in infected dog, fox or wild canine feces. *Echinococcus* parasites have a complex, two-host lifecycle (such as in dogs and sheep) in which humans are an aberrant, dead-end host. The vast majority of cases of human echinococcosis occur outside of the United States (US); however, cases within the US do occur. In this study, the authors examined death certificate data of US residents from 1990–2007 in which echinococcosis was listed as one of the diagnoses at death. The analysis demonstrated 41 echinococcosis-related deaths over the 18-year study period with foreign-born persons accounting for the majority of the deaths. This study helps quantify echinococcosis deaths among US residents and adds further support to the importance of funding echinococcosis prevention research.

## Introduction

Human echinococcosis, a neglected disease caused by the larval stages of the cestode parasites *Echinococcus granulosus* (hydatid cyst disease) and *E. multilocularis* (alveolar hydatid disease), affects an estimated 2–3 million people and results in an annual monetary loss of over $750,000,000 worldwide [1], [2]. The infection is a zoonosis, normally maintained in dogs and sheep in close association with humans (*E. granulosus*), or in a wild cycle, such as in foxes or wild canines and rodents (*E. multilocularis*). Humans are an aberrant intermediate host for the disease, developing life-threatening tissue cysts, predominately in the liver and lung, following accidental ingestion of eggs in infected dog, fox and wild canine feces. Once a human is infected, treatment is complicated and carries significant risks. Approaches include the use of antihelmintic drugs, surgery, and/or medico-surgical procedures such as PAIR (puncture, aspirate, injection of a scolicide, re-aspirate) [3].

Hydatid cyst disease, accounting for over 95% of human echinococcosis, predominates in poor, pastoral communities that raise sheep and other livestock, and keep dogs for guarding and herding because of the complex two-host lifecycle [4]. Although alveolar hydatid disease is less common in humans, it is more pathogenic, more difficult to treat, and has a higher mortality than cystic hydatid disease. Although the majority of the disease burden is located outside of the United States, both *E. granulosus* and *E. multilocularis* are endemic to regions of the United States [5]–[9]. *E. granulosus* (senso stricto or G1 strain) has been recorded in a few foci such as certain communities in Utah and California and *E. granulosus* (G8 or G10 strains) is more common in Alaska than the lower 48 states. *E. multilocularis* is endemic in wildlife across much of the mid-western United States and Alaska. In the United States, human echinococcosis is not a nationally notifiable infectious condition [10].

Despite the occurrence of human echinococcosis in the United States and its potential for causing fatal disease, there is a lack of information about echinococcosis-related deaths in the United States. Information on echinococcosis mortality is important to better understand the burden of disease and evaluate the effectiveness of public health interventions. Population-based mortality data has been used to investigate other infectious diseases, but these data have not yet been used in published echinococcosis research. We examined national mortality data to assess the burden and demographics of echinococcosis-related mortality in the United States from 1990 to 2007.

## Methods

De-identified, publicly available multiple-cause-of-death data from US death certificates from the National Center for Health Statistics (NCHS) were analyzed for the years 1990–2007 [11]. These death certificates contain basic demographic information for each decedent, including age, sex, race/ethnicity, and state of residence. In addition to designating underlying causes, the physician or coroner completing the death certificate may list up to 20 conditions that are believed to have contributed in some way to the death of an individual. Each of these conditions is coded on the basis of the *International Classification of Diseases* (ICD) system for the year in which the death occurred (*ICD*, *Ninth Revision* [ICD-9] for the period 1990–1998 and *ICD*, *Tenth Revision* [ICD-10] for the period 1999–2007).

We defined echinococcosis-related cases as US resident deaths having an ICD-9 code of 122.0–122.9 or an ICD-10 code of B67.0–B67.9 listed as an underlying or contributing cause on the death record.

Mortality rates and 95% confidence intervals (CIs) were calculated using bridged-race population estimates derived from US census data and were subsequently age-adjusted with weights from the 2000 US standard population data. Mortality rates for race/ethnicity (non-Hispanic white, non-Hispanic black, Hispanic, Asian/Pacific Islander, and Native American), sex, age, year, and state were calculated with aggregated data from all years of our study to ensure stable rates. All calculations were performed with SAS software, version 9.2.

## Results

We identified 41 echinococcosis-related deaths among US residents during the period 1990–2007 (Table 1). Age-adjusted mortality rates were higher in males (0.012 per 10^6^) than in females (0.005 per 10^6^), with males more than 2 times as likely to die from echinococcosis than were females (adjusted rate ratio = 2.2, 95% CI 1.3–3.9). Native Americans (0.062 per 10^6^), Asian/Pacific Islanders (0.032 per 10^6^), and Hispanics (0.014 per 10^6^) had the highest age-adjusted echinococcosis mortality rates, with adjusted rate ratios of 8.9 (95% CI 6.1–12.9), 4.6 (95% CI 3.2–6.7), and 1.9 (95% CI 1.3–2.9), respectively, compared to whites. No echinococcosis-related deaths were recorded in non-Hispanic blacks. The majority of echinococcosis-related deaths (35, 85%) occurred in persons over 35 years of age, with the highest rates noted in persons 85+ years. *Echinococcus* species was unspecified in 36 (88%) cases and was identified as *E. granulosus* and *E. multilocularis* in 3 (7%) and 2 (5%) cases, respectively. Site of infection was unspecified in 23 cases (56%), infection of liver and lung were recorded in 17 (42%) and 1 (2%) cases, respectively. Echinococcosis-related deaths fluctuated throughout the 18-year study period, ranging from 0–5 deaths annually, with 26 (63%) and 15 (37%) cases reported in the first and second halves of the study period, respectively. Twenty-three states reported echinococcosis-related fatalities, with California (9, 22%) having the highest number of deaths. A majority of echinococcosis-related deaths (30, 73%) occurred in foreign-born persons. Mean age at death differed slightly between foreign-born (57.9) and U.S.-born persons (64.5), and gender differences were observed (70% and 45% males, respectively).

**Table 1:** Echinococcosis-related deaths, adjusted mortality rates and rate ratios, United States, 1990–2007.

Variable	No. of deaths	Age-adjusted* mortality rate per 1,000,000 person years (95% CI)	Rate Ratio (95% CI)
All echinococcosis-related deaths	41	0.008 (0.006–0.011)	
Sex			
Male	26	0.012 (0.007–0.017)	2.2 (1.3–3.9)
Female	15	0.005 (0.003–0.008)	referent group
Race			
Non-Hispanic white	28	0.007 (0.004–0.010)	referent group
Hispanic	7	0.014 (0.002–0.025)	1.9 (1.3–2.9)
Asian/Pacific Islander	4	0.032 (0.000–0.067)	4.6 (3.2–6.7)
Native American	2	0.062 (0.000–0.154)	8.9 (6.1–12.9)
Non-Hispanic black	0		
Year			
1990	5	0.021 (0.002–0.039)	
1991	2	0.008 (0.000–0.020)	
1992	3	0.013 (0.000–0.028)	
1993	2	0.009 (0.000–0.021)	
1994	5	0.020 (0.002–0.038)	
1995	5	0.019 (0.002–0.035)	
1996	4	0.016 (0.000–0.032)	
1997	0		
1998	0		
1999	2	0.007 (0.000–0.018)	
2000	2	0.007 (0.000–0.017)	
2001	2	0.007 (0.000–0.017)	
2002	5	0.018 (0.002–0.033)	
2003	1	0.003 (0.000–0.010)	
2004	1	0.004 (0.000–0.011)	
2005	0		
2006	1	0.003 (0.000–0.009)	
2007	1	0.003 (0.000–0.010)	
Age			
<1	0		
1–4	0		
5–14	1	0.001 (0.000–0.004)	
15–24	4	0.006 (0.000–0.011)	
25–34	1	0.001 (0.000–0.004)	
35–44	6	0.004 (0.001–0.007)	
45–54	2	0.003 (0.000–0.007)	
55–64	10	0.022 (0.009–0.036)	
65–74	5	0.015 (0.002–0.028)	
75–84	5	0.023 (0.003–0.044)	
85+	7	0.094 (0.024–0.016)	

*- for sex, race, and year.

## Discussion

Although uncommon, fatal echinococcosis occurs in US residents and disproportionally affects selected demographic groups. Mortality rates were highest in males, Native Americans, Asians/Pacific Islanders, Hispanics, and persons 75 years of age and older. There was a weak declining temporal trend over the study period, and almost a quarter of all echinococcosis-related deaths occurred in residents of California. Foreign-born persons accounted for the majority of echinococcosis-related deaths; however, almost half of deaths in whites and both deaths in Native Americans were among US-born individuals.

The apparent increased risk of echinococcosis-related mortality in males may reflect higher rates of occupational exposures from the tending of livestock. The increased mortality risk in Asians/Pacific Islanders and Hispanics, all of whom were foreign born, presumably reflects greater exposure to *Echinococcus* species in their country of origin prior to emigration and upon return trips to visit friends and relatives. The observed increased risk of echinococcosis in persons over 75 years of age may be a result of increased prevalence of comorbid diseases and declining immune function. The higher number of echinococcosis-related deaths in California is expected, given the large population and sizable number of immigrants from Latin America and Asia.

Liver was the most common reported site of infection, which is expected, as liver involvement is the most common manifestation of echinococcosis. Incomplete information and lack of sufficient numbers precluded an assessment of possible associations between demographic factors and site of infection.

The occurrence of echinococcosis-related deaths in US-born persons reflects either local *Echinococcus* species transmission or travel-related infection. Because death certificates do not have information on travel, we are unable to determine whether US-born decedents acquired infections domestically or abroad.

Several important limitations are associated with the use of multiple-cause-of-death data that require consideration. Although these data are population based and contain large numbers of observations, death certificates likely underreport causes of death and may contain errors, which have been attributed to a variety of factors [12]. Mortality rates may be distorted because of errors in population estimates, particularly for race/ethnicity. Because estimates of the at-risk population factor into the denominator for rate calculations, such errors can lead to biased estimates. In addition, the small number of echinococcosis-related deaths and the lack of species-specific information make interpretation difficult. Finally, although inferential statistics are not designed for use with population-based data, we have used such methods to demonstrate that error does exist. We urge caution in the strict interpretation of our values.

Fatal echinococcosis may be more common in the United States than currently appreciated. Echinococcosis causes a mortality burden in the United States that may be modified by increased prevention and control efforts, including vaccine development for adult cestode carriers and livestock [13]. Given the presence of echinococcosis mortality in US-born persons, and the risk of travel-related exposure, hygiene precautions should be advised for individuals traveling to *Echinococcus* species endemic areas. Clinicians should be aware of the diagnosis, particularly in foreign-born patients from *Echinococcus* endemic areas, and should consider tropical infectious disease consultation early.
